# Coverage validation survey of preventive chemotherapy for schistosomiasis in interruption of transmission districts of Ethiopia, 2025

**DOI:** 10.1186/s40249-026-01439-4

**Published:** 2026-04-03

**Authors:** Chuchu Churko, Alemayehu Bekele Kassahun, Zerihun Zerdo, Tesfahun Bishaw, Nisan Kesete, Belachew Bokicho, Bisrat Molla, Gashaw Dubale, Hailemariam Atinaf, Abinet Gebremickael

**Affiliations:** 1 Collaborative Research and Training Center for Neglected Tropical Diseases, College of Medicine and Health Sciences, Arba Minch University, Arba Minch, Ethiopia; 2 Department of Medical Laboratory Sciences, College of Medicine and Health Science, Arba Minch, Ethiopia; 3 Neglected Tropical Diseases unit, Diseases prevention and control lead executive office Ministry of Health, Addis Ababa, Ethiopia; 4 Neglected Tropical Diseases Advocacy, Learning and Action Foundation, Addis Ababa, Ethiopia; 5 Neglected Tropical Diseases Program, South Ethiopia Region Health Bureau, Jinka, Ethiopia; 6 Department of Geography and Environmental Sciences, College Social Science and Humanities, Arba Minch University, Arba Minch, Ethiopia

**Keywords:** Coverage validation survey, Praziquantel, Preventive chemotherapy, Ethiopia

## Abstract

**Background:**

Ethiopia bears a substantial burden of neglected tropical diseases, including schistosomiasis, which remains a major public health problem in endemic areas. Preventive chemotherapy is the main strategy for controlling and eliminating schistosomiasis. This study aimed to assess the reach of the mass drug administration program and validate the reported praziquantel coverage in interruption-of-transmission districts of Ethiopia.

**Methods:**

A community-based cross-sectional survey was conducted from 1 to 30 August 2025 in eight purposively selected districts across three Ethiopian regions targeted for schistosomiasis interventions. Using a three-stage sampling method, data were collected using WHO-standard tools via KoboToolbox. The data were analyzed using descriptive statistics, including frequencies and percentages, in SPSS version 26 to estimate program reach and survey coverage.

**Result:**

The average programs reach (participants who offered praziquantel) was 84.2%, while surveyed coverage (participants who reported swallowing the drug) was 83.0% (95% *CI:* 82.7–83.3%). West Badewacho district achieved the highest survey coverage, 95.4%, while Hammer and Bena Tsemay districts achieved 92.1% and 92.4%, respectively. The lowest coverage was recorded in Menit Goldya (66.2%), Mizan (72.0%), and South Bench (72.4%). Treatment uptake among those offered praziquantel was 98.5%.

**Conclusions:**

The survey showed that praziquantel treatment coverage was below the 95% target and the national reported average, with notable variation across districts. Surveyed coverage was largely influenced by whether the treatment was offered; districts with lower program reach consistently had lower treatment coverage. Improving the delivery and offering of praziquantel at the community level is therefore essential to increase treatment uptake.

## Background

Neglected tropical diseases (NTDs) remain a major public health challenge in many low- and middle-income countries, particularly in sub-Saharan Africa [1, 2]. Ethiopia carries one of the largest NTD burdens globally, with millions of people affected by multiple infections that cause chronic disability, disfigurement, and reduced productivity [3]. These diseases disproportionately affect populations living in poverty, where limited access to safe water, sanitation, and healthcare services increases vulnerability to infection [1, 4]. In Ethiopia, twelve NTDs are prioritized for control, elimination, or eradication: schistosomiasis (SCH), soil-transmitted helminthiasis (STH), trachoma, onchocerciasis, lymphatic filariasis, leishmaniasis, podoconiosis, Guinea worm disease (dracunculiasis), scabies, rabies, leprosy, and arboviral infections such as dengue and chikungunya [3, 5]. Many of these diseases are co-endemic in the same districts, creating overlapping health burdens and requiring integrated programmatic responses [3].

Schistosomiasis remains one of the most important NTDs in Ethiopia and continues to pose a substantial public health problem [2, 3]. The disease is caused by trematode parasites of the genus *Schistosoma*, primarily *Schistosoma mansoni* and *S. haematobium*, and is transmitted through contact with freshwater contaminated with cercariae released by infected snail intermediate hosts [2, 6]. Infection often occurs during routine activities such as swimming, bathing, fishing, irrigation, or washing clothes in rivers and lakes [6]. Consequently, communities living near freshwater bodies and those engaged in agricultural activities are particularly vulnerable [2]. Chronic infection may result in significant morbidity, including hepatosplenic disease, anemia, growth retardation, and impaired cognitive development among children [2, 7].

The epidemiology of schistosomiasis in Ethiopia shows substantial geographic variation, largely influenced by ecological conditions, altitude, water resource development, and sanitation coverage [3, 8]. It is estimated that more than 5 million people are infected with schistosomiasis in the country, while over 37 million people live in areas at risk of infection [3, 8]. The disease is widely distributed across several regions, particularly in areas where environmental conditions favor the survival of intermediate snail hosts and frequent human-water contact occurs [8]. Epidemiological studies conducted in different parts of the country have reported considerable variability in prevalence, ranging from low levels in some communities to very high levels in highly endemic areas [8]. School-aged children are often the most affected group due to their frequent exposure to contaminated water sources [2, 6].

In response to the substantial burden of schistosomiasis and other helminth infections, Ethiopia conducted nationwide mapping of schistosomiasis and soil-transmitted helminthiasis between 2013 and 2015 to better understand the distribution and intensity of infection [3]. The mapping results identified a large number of endemic districts requiring preventive chemotherapy interventions and informed the development of a national NTD control strategy [3, 5]. Following this mapping exercise, the Federal Ministry of Health launched a large-scale national deworming program targeting at-risk populations in endemic areas [5].

Preventive chemotherapy with praziquantel (PZQ) is the primary strategy recommended by the World Health Organization for controlling schistosomiasis in endemic settings [6]. In Ethiopia, preventive chemotherapy is delivered through mass drug administration (MDA) campaigns implemented via school-based and community-based distribution platforms [5]. These campaigns target at-risk populations, particularly school-aged children, and are conducted periodically depending on the level of endemicity within each district [6]. Since the introduction of nationwide MDA, millions of individuals have received PZQ annually, contributing to reductions in infection prevalence and intensity in several endemic areas [5].

Evidence from program evaluations and operational studies suggests that repeated rounds of MDA have contributed to measurable reductions in schistosomiasis prevalence and infection intensity in some parts of the country [9]. However, transmission persists in many endemic areas due to ongoing exposure to contaminated water, inadequate water and sanitation infrastructure, and variations in program implementation and treatment coverage [2, 6]. These challenges highlight the need for sustained interventions and effective monitoring systems to track progress toward control and elimination targets [6].

A critical component of NTD program monitoring is the assessment of preventive chemotherapy coverage, defined as the proportion of the eligible population who actually receive and swallow the distributed drugs [10]. Accurate measurement of treatment coverage is essential because program managers rely on these data to evaluate program performance and determine whether coverage targets have been achieved [10]. However, routine administrative coverage reports may be affected by reporting errors, incomplete treatment registers, or inaccurate population estimates, which can lead to overestimation or underestimation of true program coverage [10].

Coverage validation surveys are therefore conducted to independently verify reported MDA coverage [10]. These surveys provide more reliable estimates by collecting information directly from community members about drug receipt and consumption, thereby minimizing biases associated with routine program data [10]. The validated survey results are then compared with reported coverage obtained from treatment records at school, community, district, and regional levels to assess the accuracy of program reporting and to identify potential gaps in program implementation [10].

To accelerate progress toward elimination, Ethiopia has intensified its efforts through the Interruption of Transmission (IoT) initiative, which aims to halt schistosomiasis transmission in low-prevalence districts through intensified interventions, including repeated MDA, enhanced surveillance, and improved water, sanitation, and hygiene (WASH) measures [4]. Monitoring treatment coverage within these districts is essential to ensure that MDA campaigns achieve adequate reach and contribute to interruption of transmission [4]. In this context, an integrated post-MDA coverage validation survey was conducted in selected IoT districts to assess the reach of the mass drug administration program and to validate the reported PZQ treatment coverage. The findings of this survey are intended to provide evidence for improving program implementation and informing future MDA rounds aimed at interrupting schistosomiasis transmission in Ethiopia.

## Methods

### Study area and period

**Fig. 1:**
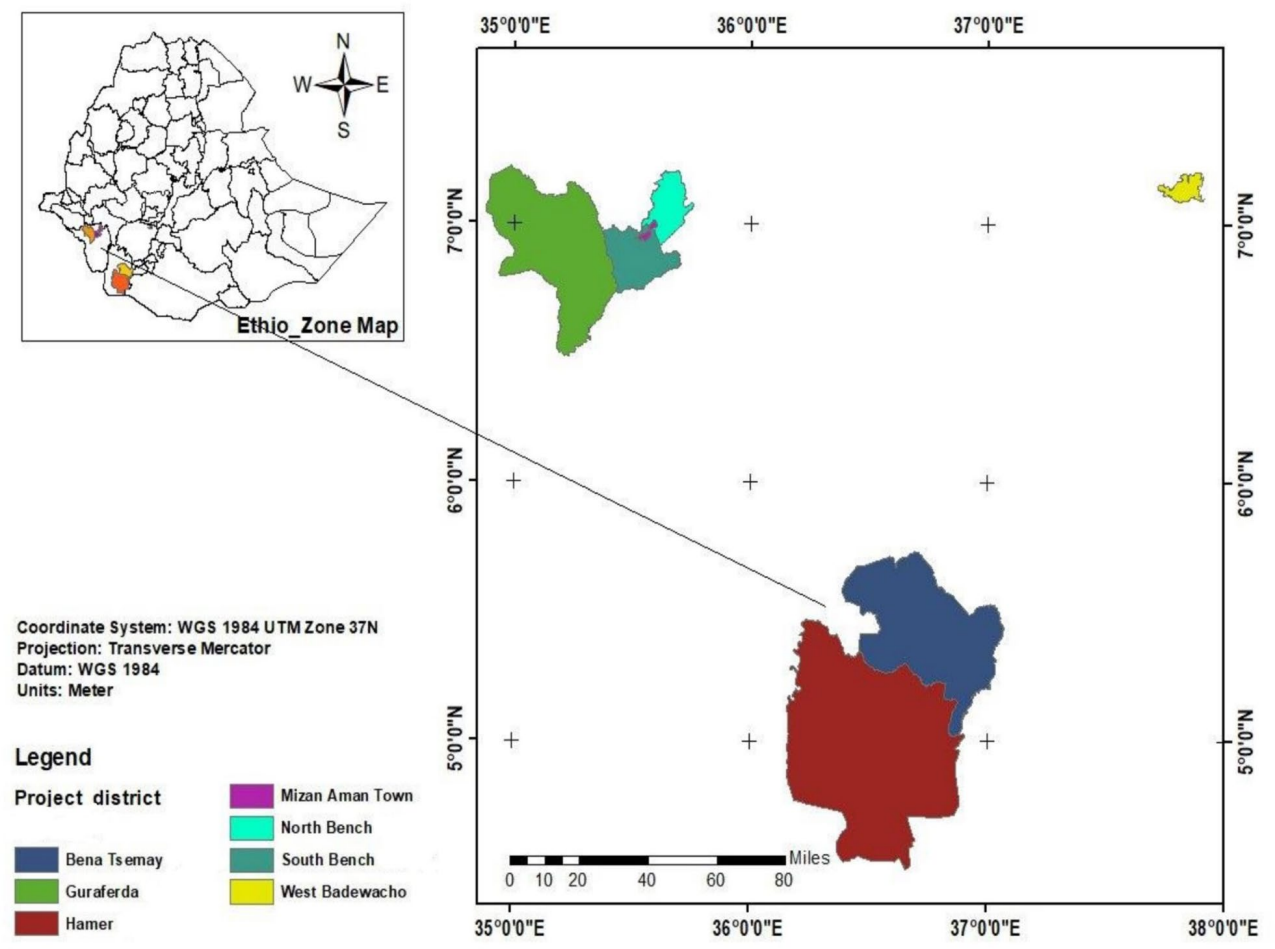
Name & location of the IOT districts of Ethiopia, 2025

This coverage validation survey was conducted in eight districts across three regions of Ethiopia: Southern, Central, and Southwestern Ethiopia. The IoT districts were purposively selected prior to the coverage survey, based on a list provided by Neglected Tropical Diseases Advocacy, Learning and Action (NALA) and the Ministry of Health (MoH). These districts were selected because their relatively low schistosomiasis endemicity made them suitable candidates for transmission interruption interventions. Two districts from the South Ethiopia region (Hammer and Bena Tsemay), five districts from the South West Ethiopia Region (Mizan, Guraferda, South Bench, Meinit Goldiya, and North Bench), and one district (West Badewacho) from the central Ethiopia region were included in the survey (Fig. 1). Data were collected from 1 to 30 August 2025.

### Study design and study population

A community-based cross-sectional study design was applied. All school-age children (5–14 years) and adults aged 15 and above were included as the target population for PZQ treatment across all districts.

### Data collection procedure

**Fig. 2:**
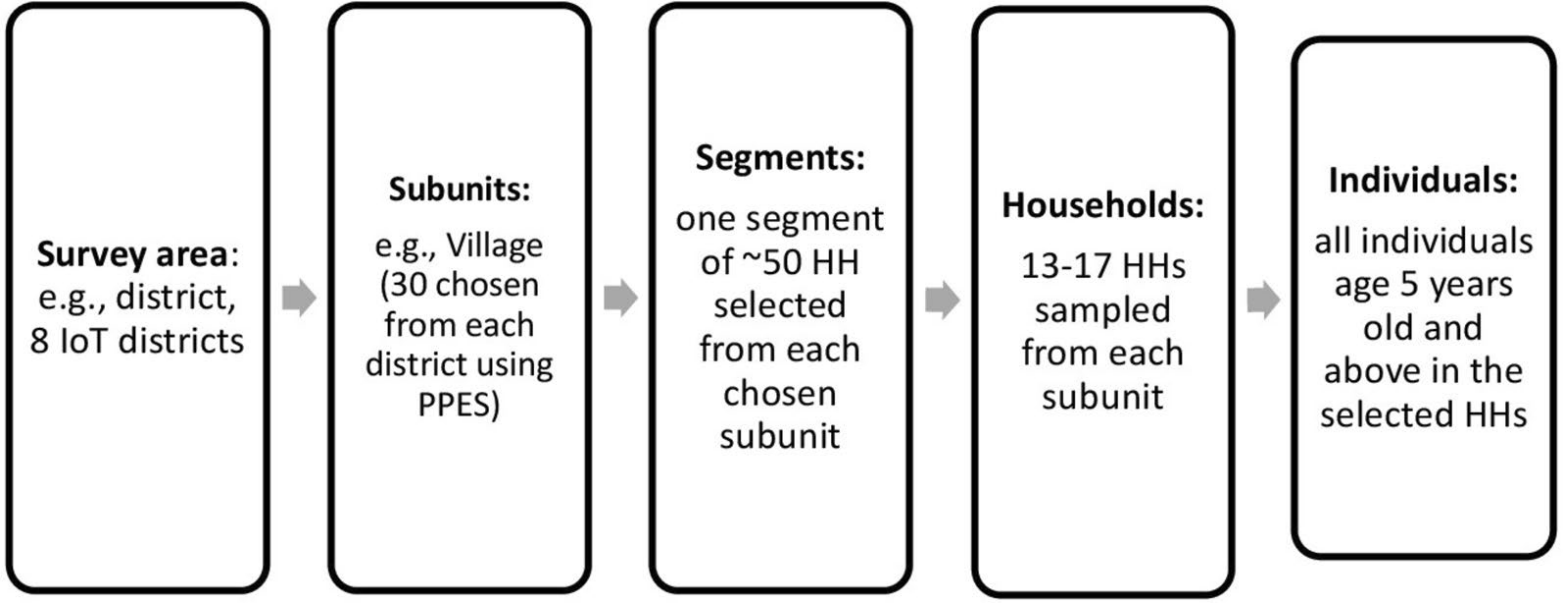
Schematic presentation of sample procedure in the study area

A team of four data collectors and supervisors was deployed to the respective IoT districts. Upon arrival, the teams conducted interviews with the heads of randomly selected villages to gather relevant village data and prepared segments of the selected sub-units, typically kebeles. Within these randomly selected segments, households with eligible participants were selected using List-A and List-B generated by the survey sample builder. Household and individual interviews were then conducted. The data collection process utilized the standard World Health Organization (WHO) tool and was carried out using KoboToolbox for digital data entry [11]. The reported coverage of each district was obtained from the Minsitry of Health, Ethiopia and implementing partner, NALA. The reported coverage (100.4%) refers to the administrative coverage obtained from the routine program reports of the mass drug administration campaign (Fig. 2).

### Data quality control

Before the actual data collection, the data collectors underwent 2 days of intensive training on the survey objectives and data collection protocols. The training covered the study objectives, data collection tools, and standardized procedures. Participants were also instructed on identifying and addressing potential field challenges, maintaining data quality, upholding ethical standards, and communicating effectively with study participants. This preparation ensured that data collection was accurate, reliable, and conducted ethically throughout the survey. The training also included a practical component, where the team conducted a mock survey at the community level in Arba Minch town to test the data collection tool. A data quality monitoring team was established at the central level to oversee the data collection process. This team monitored data quality from the first day of data collection, providing timely feedback to the data collectors and supervisors to ensure accuracy. To guarantee data collection from all eligible households and ensure comprehensive geographic coverage within the selected sub-units, GPS coordinates were mapped and evaluated. Recall bias was minimized by presenting sample PZQ tablets to the interviewees. Additionally, data collection was conducted within 2 weeks of the MDA campaign to enhance recall accuracy.

### Data processing and analysis

Data were analyzed using IBM SPSS Statistics version 26 (IBM Corp., Armonk, NY, USA). Program reach was calculated as the total number of respondents that responded “Yes” to being offered the drug from the total number of people surveyed:

Programme reach = Number of “yes” responses to offered the drug/Total number of people surveyed

Whereas, survey coverage was calculated as the total number of participants that responded “Yes” to swallowing the drug from the total number of people surveyed.

Survey coverage = Number of “yes” responses to having swallowed the drug/Total number of people surveyed.

No inferential statistical tests were performed, and no significance level was set, as the analysis focused solely on describing coverage and other program indicators. Results were presented in text, tables, and figures to clearly show program coverage and other key indicators.

## Results

Of the total 10,490 individuals, 9595 participated in the survey, representing a response rate of 91.5%. Participation varied across districts, with the highest number of respondents recorded in Bena Tsemay District (*n* = 1612) and the lowest in Guraferda District (*n* = 259). In terms of demographic characteristics, the majority of respondents were adults aged 15 years and above, accounting for 72.0% of the total sample (Table 1). The mean age of the study participants was 25.9 with standard deviation of ± 14.9.

**Table 1:** Socio-demographic characteristics of the study subjects for praziquantel uptake, *n* = 9595

Variables	*n*	Proportion (%)
Name of districts		
Bena Tsemay	1612	16.8
Guraferda	259	2.7
Hammer	1309	13.6
Menit Goldiya	1272	13.2
Mizan	1277	13.3
North Bench	1004	10.5
South Bench	1263	13.2
West Badewacho	1599	16.7
Age category		
15 and above years old	6941	72.3
5–14 years old	2654	27.7
Sex		
Female	4782	49.8
Male	4813	50.2
Occupation		
Farmer	2486	25.9
Pastoralist	901	9.4
Others*	102	1.0
Housewife	1590	16.6
Student	2754	28.7
Civil Servant	354	3.7
Self-employed	402	4.2
Unemployed	1006	10.5
If student, currently attending school, (*n* = 2754)		
No	147	5.3
Yes	2607	94.7
If student, level of education, (*n* = 2754)		
Primary	1936	74.3
Secondary	511	19.6
Higher education or technical and vocational	86	3.3
Kindergarten	74	2.8

^*^Others: religious leaders, not schooling children, Fisherman

### Community awareness about the MDA campaign

Among the respondents interviewed, 79% reported that they were aware of the PZQ MDA campaign prior to the actual date of drug distribution. The primary sources of information were health professionals (68.4%), followed by teachers (11.7%) and places of worship (10.8%).

Regarding specific knowledge about the campaign, 70.4% of participants knew the exact location where the tablets would be distributed, while 55.4% were aware of the disease that the tablets are intended to treat (Table 2).

**Table 2:** Community awareness towards MDA activities in IoT districts of Ethiopia, 2025 (*n* = 9595)

Variables	*n*	Proportion (%)
Received information about MDA activities that took place in June, 2025 (*n* = 9595)		
No	2016	21
Yes	7579	79
If Yes, from whom did you receive? (*n* = 7579)		
Teacher	884	11.7
Village meeting	988	13
Flyer/Banner	54	0.7
Health professionals	5185	68.4
Mass media	112	1.5
Load speaker	561	7.4
Place of worship	294	3.9
Parents	820	10.8
Neighbors	702	9.3
Others	105	1.4
Received any information about (*n* = 7579)		
The dates of MDA	3751	49.5
Place where the tablets would be distributed	5339	70.4
The diseases that these tablets can treat	4201	55.4
The side effects people may feel after taking tablets	1484	19.6
If you need to eat before taking the tablets	2979	39.3
Who was eligible to receive the tablets	1901	25.1
Received Explanation about the benefits of taking tablets (*n* = 9595)		
No	3520	36.7
Yes	6075	63.3
If yes, what did you receive (*n* = 6075)		
Curing SCH	3414	56.2
Curing STH	5882	96.8
Improving school performance	167	2.7
Growing stronger/healthier	172	2.8
Preventing cancer	21	0.3
Preventing infertility	15	0.2
Heard any negative reports or concerns from any source (*n* = 9595)		
No	9095	94.8
Yes	500	5.2
If yes, what are these reports/concern (*n* = 500)		
death	17	3.4
Nausea	348	69.6
Dizziness	180	36.0
Mental disability	9	1.8
Physical disability	13	2.6
Sources of negative reports or concerns (*n* = 500)		
Community	260	52.0
Health workers	5	1.0
Parents	94	18.8
School friends	112	22.4
Self	29	5.8
Knows signs and symptoms of SCH (*n* = 9595)		
No	7652	79.7
Yes	1943	20.3
If Yes, what are these signs and symptoms (*n* = 1943)		
Blood in urine	208	10.7
Blood in the stool	450	23.2
Abdominal pain	1270	65.4
Painful urination	343	17.7
Frequent urination	168	8.6
Swollen stomach	785	40.4
Nausea/vomiting	701	36.1
Knew prevention measures (*n* = 9595)		
No	6864	71.5
Yes	2731	28.5
If yes, what are the prevention measures (*n* = 2731)		
Taking tablets during MDA	2064	75.6
Visiting health facilities	1184	43.4
Using toilets	908	33.2
Avoid contact with contaminated/stagnant water	1219	44.6
Use well or pump water	395	14.5
Treat water before consumption	599	21.9
Others*	65	2.4

Others*: Use herbal medicine, keep personal hygiene, and avoid eating of raw milk and meat, wearing shoes; *MDA* Mass drug administration, *IoT* Interruption of transmission, *SCH* Schistosomiasis, *STH* Soil transmitted helminthiasis

### PZQ treatment coverage

The surveyed coverage of PZQ among the eligible population was 84.2% (95% *CI:* 83.9–84.5%). Coverage varied notably between districts, with the highest uptake recorded in West Badewacho district (95.4%) and the lowest in Menit Goldya (66.2%). Among those who were offered the drug during the MDA campaign, and 98.5% of the respondents swallowed the drug (Table 3).

**Table 3:** Surveyed coverage of praziquantel in the IoT district of Ethiopia (*n* = 9595)

Variables	Total	Offered, *n* (%)	Surveyed coverage, *n* (%)	Coverage among offered, %
Name of districts				
Benatsemay	1612	1490 (92.4)	1489 (92.4)	99.9
Guraferda	259	224 (86.5)	223 (86.1)	99.6
Hammer	1309	1205 (92.1)	1205 (92.1)	100.0
Menit Goldiya	1272	863 (67.8)	842 (66.2)	97.6
Mizan	1277	997 (78.1))	920 (72.0)	92.3
North Bench	1004	844 (84.1)	843 (84.0)	99.9
South Bench	1263	916 (72.5)	915 (72.4)	99.9
West Badewacho	1599	1542 (96.4)	1526 (95.4)	99.0
Overall, *n* (%), 95% *CI*	9595	8081 (84.2) (83.9–84.5)	7963 (83.0) (82.7–83.3)	98.5
Age category				
15 and above years old	6941	5844 (84.2)	5737 (82.7)	98.2
5–14 years old	2654	2237 (84.3)	2226 (83.9)	99.5
Sex				
Female	4782	3959 (82.8)	3874 (81.0)	97.9
Male	4813	4122 (85.6)	4089 (85.0)	99.2
Occupation				
Farmer	2486	2080 (83.7)	2066 (83.1)	99.3
Pastoralist	901	840 (93.2)	840 (93.2)	100.0
Others	102	75 (73.5)	75 (73.5)	100.0
Housewife	1590	1265 (79.6)	1214 (76.4)	95.9
Student	2754	2411 (87.5)	2385 (86.6)	98.9
Civil servant	354	317 (89.5)	305 (86.2)	96.2
Self-employed	402	330 (82.1)	326 (81.1)	98.8
Unemployed	1006	763 (75.8)	752 (74.8)	98.6
Received any information about the June MDA activities				
No	2016	1127 (55.9)	1102 (54.7)	97.8
Yes	7579	6954 (91.8)	6861 (90.5)	98.7
Heard any negative reports/concerns regarding June, MDA				
No	9,095	7,629 (83.9)	7,523 (82.7)	98.6
Yes	500	452 (90.4)	440 (88.0)	97.3
Knew signs and symptoms of SCH				
No	7,652	6,278 (82.0)	6,176 (80.7)	98.4
Yes	1,943	1,803 (92.8)	1,787 (92.0)	99.1
Knew prevention measures				
No	6864	5473 (79.7)	5375 (78.3)	98.2
Yes	2731	2608 (95.5)	2588 (94.8)	99.2

*MDA* Mass drug administration, *IoT* Interruption of transmission, *SCH* Schistosomiasis

### Comparing surveyed coverage with reported and IoT threshold coverage

**Fig. 3:**
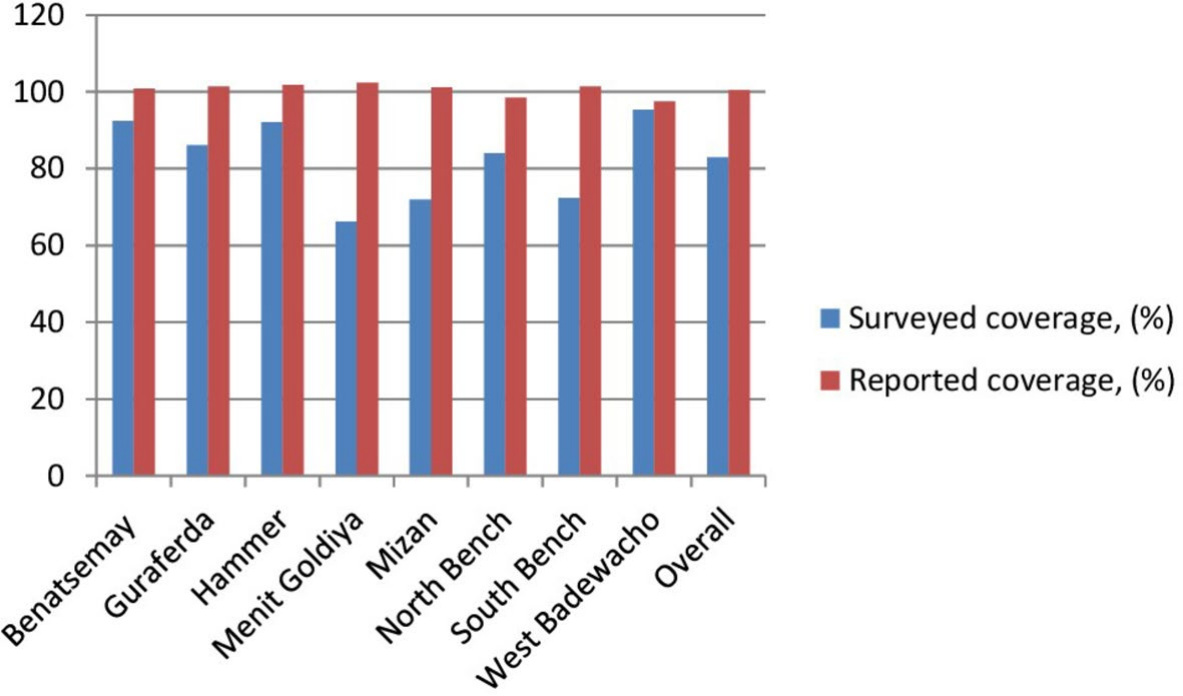
Comparison of reported and surveyed coverage in IoT districts of Ethiopia, 2025

The overall surveyed coverage was 83.0% (95% *CI:* 82.7–83.3%) which was significantly lower than the reported coverage average of 100.4%. Except West Badewacho none of the surveyed districts met the national target of treating at least 95% of the IOT target population, as reflected in the reported coverage by districts. The Menit Goldya district had a notably lower surveyed coverage (66.2%) compared to the other surveyed districts (Fig. 3).

**Fig. 4:**
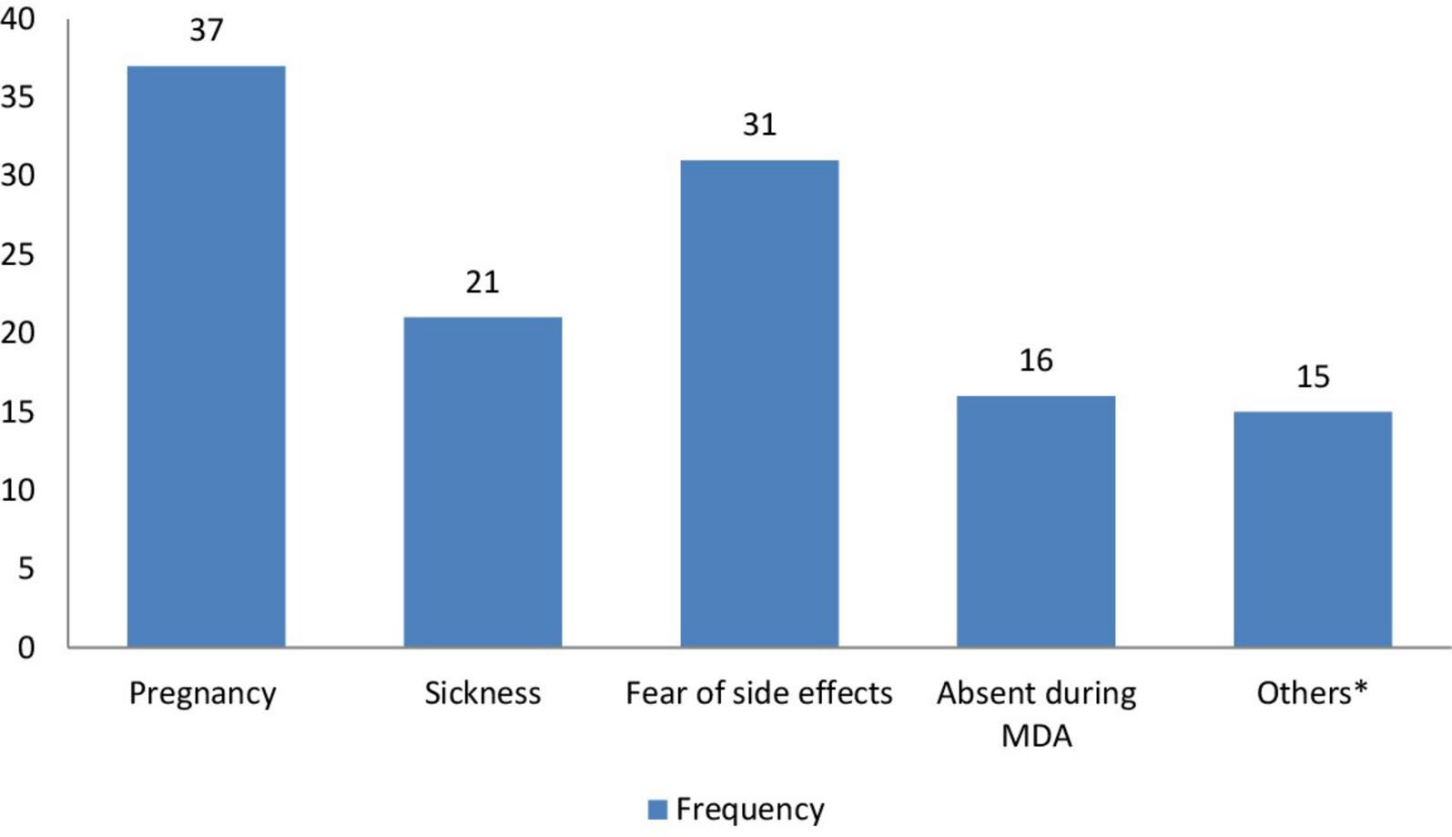
Reasons for not swallowing the PZQ tablet in IOT districts, Ethiopia, 2025. Others*: breastfeeding, refused to take, lack of awareness about drugs, HEWs not willing to bring the drug, being careless

A total of 118 study subjects did not swallow the PZQ tablets provided during the MDA. The most frequently reported reason was 37 (31.4%) reported being pregnant at the time of MDA, followed by 31 (26.3%) reported fear of side effects (Fig. 4).

### Mass drug administration distribution sites

**Fig. 5:**
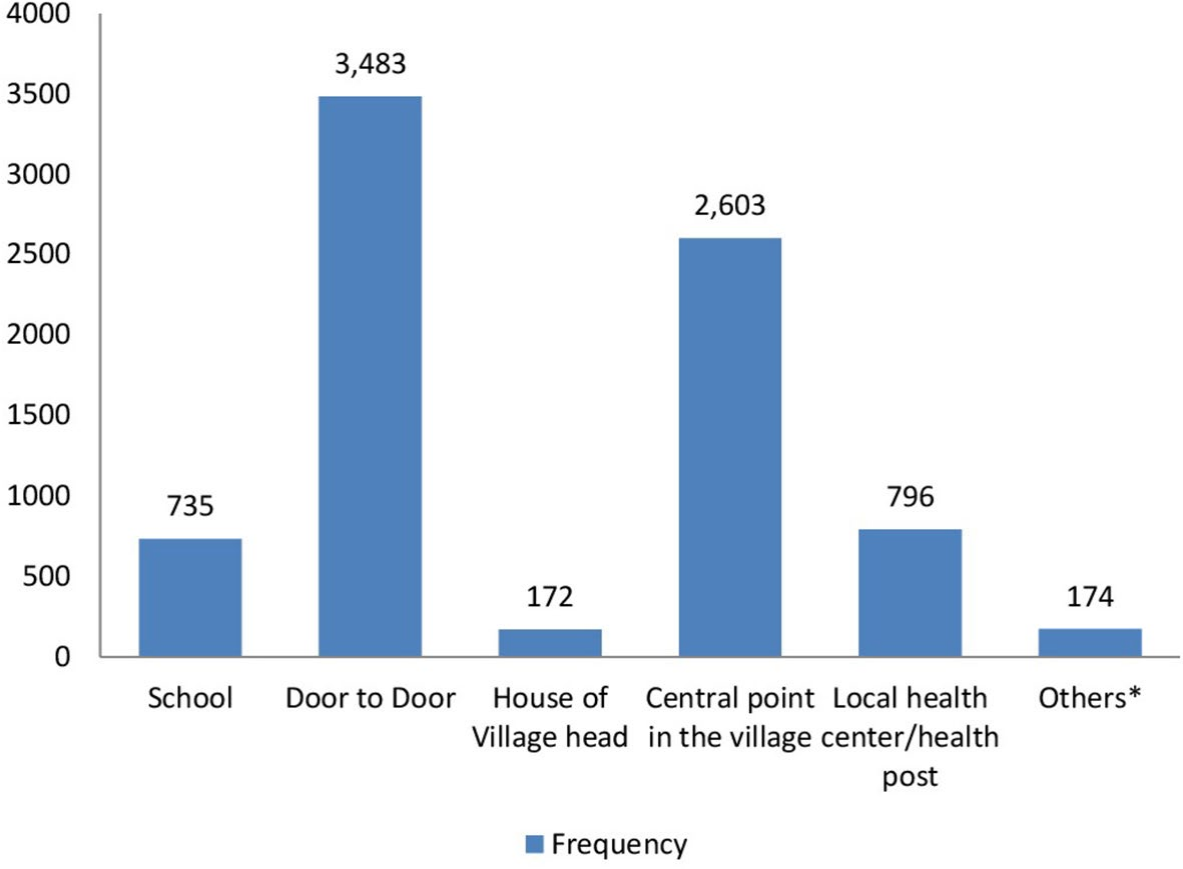
Places where the survey participants took the MDA drugs, Ethiopia, 2025

Of the 7963 participants who swallowed the drugs, the majority, 3483 (43.7%) receiving it through the door-to-door campaign approach, making it the most commonly utilized method. This was followed by distribution at the central point in the village (2603, 32.7%). Institutional settings contributed a smaller share, with 796 (10.0%) participants receiving the drugs at local health centers or health posts and 735 (9.2%) at schools (Fig. 5).

## Discussion

The validated survey coverage of PZQ among the eligible population was 84.2%, which is falls short of the WHO target of at least 95% in IoT districts [12] and is significantly lower than the average national reported coverage of 100.4%. Coverage varied widely across districts, reflecting differences in local implementation capacity and community engagement. West Badewacho achieved the target (95.4%), while Menit Goldya recorded the lowest coverage (66.2%), consistent with geographic inequalities reported in other Ethiopian schistosomiasis programs [8, 13]. The surveyed coverage was significantly higher than previous studies of coverage evaluation finding of PZQ (75.5%) [14]. The higher PZQ coverage observed in this study compared with previous coverage reports likely reflects both programmatic improvements and methodological differences. This survey was conducted in IoT districts, where repeated treatment rounds, targeted community sensitization, and better supervision likely increased acceptance. Additionally, improved drug availability and trained distributors, combined with greater community familiarity with treatment, likely reduced refusal and non-uptake relative to earlier studies.

Among participants who were offered the drug, nearly all (98.5%) swallowed it, indicating high compliance when the medicine is provided. Overall uptake of 84.2% reflects both the offering of the drug and its swallowing, highlighting that increasing program reach could further improve total swallowing coverage. Similar findings have been reported in national coverage validation studies where failure to reach the target population, rather than refusal, accounted for most missed treatments [15]. Reported reasons for non-compliance—pregnancy, fear of side effects, illness, and absence align with those found in other African studies [16, 17]. Targeted communication and flexible delivery mechanisms are needed to reduce these missed opportunities.

Our study found that only about half of the respondents (55.4%) were aware that PZQ is intended to treat SCH, and 56.2% knew that the drug cures the disease. This is consistent with previous findings in Ethiopia and other sub-Saharan African countries, where awareness of SCH and its treatment through MDA is often limited. Low knowledge and misconceptions about PZQ, including concerns about side effects, have been shown to reduce uptake and compliance with treatment campaigns [18, 19].

Limited awareness of schistosomiasis treatment has important programmatic implications. Studies indicate that individuals who understand the purpose of PZQ are more likely to participate in MDA campaigns, improving coverage and effectiveness of disease control [20]. Strengthening community education and health communication on SCH and PZQ treatment is therefore critical to enhance treatment acceptance and achieve sustained interruption of transmission in endemic areas [18–20].

Consistent with our findings (males 85% vs females 81%), other studies show that gender generally does not strongly affect MDA coverage, though minor differences may occur. Consistently, in a community-wide PZQ MDA in Mali, overall treatment coverage did not differ significantly between males and females, suggesting that equitable distribution strategies can achieve balanced uptake across sexes [21]. In contrast, several surveys in Ethiopia found that males were slightly more likely than females to swallow PZQ, possibly reflecting gender differences in school attendance, health knowledge, or availability during MDA activities [13]. These mixed patterns may be influenced by local social roles, time spent at home versus in the community, and how sensitization messages reach men and women differently, highlighting the importance of tailored mobilization to maintain high coverage for both genders.

Limitations of this study include potential recall bias from self-reported drug uptake and the purposive selection of districts, which may limit generalizability beyond IoT areas. Clinical assessments were not conducted, and more advanced statistical analyses were not performed, as this was a descriptive survey focused primarily on coverage. We recommend future studies include clinical evaluations and use robust designs with appropriate statistical analyses. Nonetheless, the findings provide valuable evidence to inform the design and monitoring of future MDA campaigns.

## Conclusions

While PZQ uptake among those reached was high, overall survey coverage remains below the elimination threshold and significantly lower than reported coverage. Strengthening community mobilization, expanding adult coverage, and addressing district-level disparities are essential to accelerate progress toward SCH elimination in Ethiopia. In addition, program reach should be improved through increasing availability and accessibility of the MDA drugs for the eligible populations.

## Data Availability

The datasets used and/or analysed during the current study are available from the corresponding author on reasonable request.

## Abbreviations

IOT: Interruption of transmission
IRB: Institutional Research Review Board
MDA: Mass Drug Administration
MOH: Ministry of Health
NALA: Neglected tropical diseases advocacy learning and action
NTDs: Neglected tropical diseases
PC: Preventive chemotherapy
PZQ: Praziquantel
SCH: Schistosomiasis
SPSS: Statistical package for social sciences
STH: Soil transmitted helminthiasis
WHO: World Health Organization
